# Co-Processing Versus Infusion for Turmeric–Ginger Functionalization of Olive Oil: Effects on Quality, Bioactivity, and Storage Stability

**DOI:** 10.3390/foods15213811

**Published:** 2026-10-24

**Authors:** Antonio Gattuso, Irene Maria Grazia Custureri, Vincenzo Sicari, Monica Rosa Loizzo, Angelo Maria Giuffré

**Affiliations:** 1 Department of Agraria, Mediterranea University of Reggio Calabria, Località Feo di Vito, 89124 Reggio Calabria, RC, Italy; antonio.gattuso@unirc.it (A.G.); irene.custureri@unirc.it (I.M.G.C.); amgiuffre@unirc.it (A.M.G.); 2 Department of Pharmacy, Health and Nutritional Sciences, University of Calabria, 87036 Rende, CS, Italy

**Keywords:** extra virgin olive oil, turmeric, ginger, malaxation, infusion, oxidative stability, antioxidant activity, enzyme inhibition

## Abstract

This study compared co-processing during malaxation (TG_MAL) and post-extraction infusion (TG_INF) for the functionalization of olive oil with turmeric and ginger, using untreated extra virgin olive oil (U_EVOO) as a control. Three independent batches per treatment were monitored over 360 days for quality indices, color, apparent pigments, α-tocopherol, total phenolic content (TPC), antioxidant responses, in vitro digestive-enzyme inhibition, and sensory attributes. At T360, TG_MAL had the lowest peroxide value (9.79 meq O_2_ kg^−1^), compared with TG_INF (14.03) and U_EVOO (15.82), whereas TG_INF had the highest free acidity (1.04%). Both functionalized oils retained higher TPC than the control at T360 (379.65 and 384.65 versus 309.65 mg kg^−1^ for TG_MAL, TG_INF, and U_EVOO, respectively). Antioxidant responses were assay-dependent: functionalization did not consistently improve DPPH or ABTS scavenging, although lower apparent IC_50_ values were obtained in the β-carotene bleaching assay. TG_INF showed the lowest IC_50_ values for α-amylase, α-glucosidase, and pancreatic lipase at T360, indicating greater persistence of in vitro inhibitory activity. The two protocols also produced distinct color and sensory profiles. These findings demonstrate protocol-dependent trade-offs rather than a universally superior approach. Interpretation is limited by potential spice-derived spectral interference, different nominal spice dose bases, and the additional 30-day infusion period preceding the TG_INF storage baseline.

## 1. Introduction

Extra virgin olive oil (EVOO) is a key component of the Mediterranean diet and is highly valued for its nutritional, sensory, and functional properties. Obtained exclusively by mechanical or other physical means, EVOO is characterized by a high proportion of monounsaturated fatty acids, mainly oleic acid, together with a complex fraction of minor constituents, including phenolic compounds, tocopherols, carotenoids, phytosterols, and volatile compounds [1,2,3]. These minor constituents contribute to the sensory characteristics and oxidative stability of EVOO and are also associated with several of its biological properties [2,3].

Regular consumption of EVOO, particularly within a Mediterranean diet, has been associated with beneficial cardiovascular and metabolic outcomes [4,5,6]. These effects have been partly related to the phenolic fraction of EVOO, whose constituents exhibit antioxidant and anti-inflammatory activities and may modulate pathways involved in oxidative and inflammatory processes [5,6,7].

Growing interest in foods containing naturally occurring bioactive compounds has stimulated the development of strategies for enriching conventional food matrices. Olive oil is particularly suitable for incorporating plant-derived compounds with affinity for the lipid phase. Consequently, aromatic herbs, spices, and other botanical materials have been incorporated into olive oil to modify its phytochemical composition, oxidative stability, in vitro functional properties, and sensory characteristics [8,9,10,11,12,13].

Among the botanical ingredients investigated for food applications, turmeric (*Curcuma longa* L.) and ginger (*Zingiber officinale* Roscoe), both belonging to the Zingiberaceae family, are of particular interest because of their characteristic phytochemical profiles. Turmeric is rich in curcuminoids, mainly curcumin, demethoxycurcumin, and bisdemethoxycurcumin, which have been extensively investigated for their antioxidant and anti-inflammatory properties [14,15,16]. Ginger is characterized by gingerols, shogaols, and related phenolic constituents that contribute to its characteristic pungency and have also been investigated for antioxidant, anti-inflammatory, and metabolic activities [17,18]. The combined use of turmeric and ginger may therefore represent an interesting approach for incorporating different classes of phytochemicals into the same food matrix [13].

Several technological approaches have been proposed to obtain flavored or functionalized olive oils. Post-extraction infusion or maceration involves placing plant materials in contact with the oil to promote the transfer of soluble constituents into the lipid phase. The efficiency of this process depends on several factors, including the nature and concentration of the botanical material, particle size, contact time, temperature, and chemical properties of the compounds involved [9,13]. Alternatively, plant materials can be incorporated directly into the olive paste during malaxation. This co-processing approach allows the botanical material to interact with the olive paste before oil separation and may produce oils with chemical, oxidative, and sensory characteristics that differ from those obtained by post-extraction infusion [9,10,11,13].

Previous studies have investigated olive oils enriched with ginger or turmeric and have shown that the enrichment procedure can influence phenolic content, antioxidant activity, oxidative stability, and sensory characteristics [11,12,13]. Custureri et al. [11] compared malaxation and infusion to produce ginger-enriched Ottobratica EVOO during one year of storage, whereas Custureri et al. [12] applied similar approaches to turmeric-flavored Ottobratica olive oil. Both studies showed process-dependent differences in the evolution of quality parameters, antioxidant activity, in vitro functional properties, and sensory characteristics during storage. Arslan and Acar [13] also investigated olive oils aromatized with ginger, turmeric, and their combination using co-processing and maceration and reported differences related to the enrichment procedure. However, information on the long-term behavior of olive oils simultaneously functionalized with turmeric and ginger remains limited, particularly regarding oxidative stability, antioxidant characteristics, in vitro digestive-enzyme inhibition, and sensory properties during prolonged storage.

Therefore, the aim of the present study was to compare two protocols for producing olive oils functionalized with a combination of turmeric and ginger powders: co-processing during olive malaxation and post-extraction infusion. The oils were monitored over 360 days of storage by evaluating physicochemical quality parameters; pigment content; total phenolic content; α-tocopherol; antioxidant activity; in vitro inhibitory activity against α-amylase, α-glucosidase, and pancreatic lipase; and sensory characteristics. The study was designed to characterize the chemical, oxidative, in vitro functional, and sensory evolution of the oils obtained using the two functionalization protocols and to assess their potential for the development of value-added olive oil-based products.

## 2. Materials and Methods

### 2.1. Olive Oil Production and Functionalization

Olives (*Olea europaea* L., cv. Ottobratica) were harvested at the appropriate ripening stage from an experimental orchard located in San Giorgio Morgeto (Reggio Calabria, Southern Italy; 38°23′28″ N, 16°05′11″ E). Immediately after harvesting, the fruits were transported to the laboratory in perforated high-density polyethylene (HDPE) crates (40 L capacity) to minimize mechanical damage and preserve fruit quality before processing.

Olive oil was produced on the day of harvest using a laboratory-scale extraction system (Agrimec Valpesana, Calzaiolo, San Casciano Val di Pesa, Florence, Italy). The olives were crushed to obtain a paste, which was malaxed for 40 min at room temperature (25 ± 2 °C) and subsequently subjected to hydraulic pressing. The pressure was gradually increased to a maximum of 200 atm over approximately 20 min. The oil phase was then carefully separated from the vegetation water and solid residues.

Commercial food-grade turmeric (*Curcuma longa* L.) and ginger (*Zingiber officinale* Roscoe) powders were purchased from a local retailer (Reggio Calabria, Italy). Functionalized oils were prepared using two different protocols: co-processing during malaxation and post-extraction infusion.

For the co-processing treatment, a mixture of turmeric and ginger powders (50:50, *w*:*w*) was added to the olive paste at a total concentration of 1% (*w*/*w*) immediately before malaxation. The mixture was then processed under the same conditions described above, allowing the botanical material to interact with the olive paste during oil extraction. The resulting oil was designated TG_MAL. The turmeric-to-ginger ratio (50:50, *w*/*w*) and the total concentration of 1% (*w*/*w*) were selected based on preliminary experimental trials and previous studies investigating the functionalization of olive oil with turmeric and ginger [11,12,13]. This formulation was adopted to incorporate both botanical ingredients at equal proportions while maintaining a moderate overall spice loading suitable for comparing the two functionalization protocols. At the nominal total loading of 1% (*w*/*w*), each powder accounted for 0.5% of the mass of the receiving matrix. The receiving matrix was olive paste for TG_MAL and extracted oil for TG_INF; consequently, these nominal loadings do not represent equal spice inputs per kilogram of recovered oil. The comparison concerns the complete preparation protocols rather than two processes standardized for the same final spice-derived compound concentration.

For the post-extraction infusion treatment, the same turmeric–ginger mixture was added to freshly extracted olive oil at a total concentration of 1% (*w*/*w*). The suspension was maintained under continuous magnetic stirring in sealed 500 mL amber glass flasks for 30 days at 20 ± 3 °C and protected from light. After the infusion period, the oil was separated from the solid plant material by filter paper (Whatman, pore size: 7–12 μm, Merck, Germany) and designated TG_INF.

Non-functionalized olive oil produced under the same extraction conditions was used as the control (U_EVOO). Three independent production batches were prepared for each treatment. Oil from each batch was distributed into separate bottles allocated to the different storage times. At each sampling time, a previously unopened bottle from each batch was opened and analyzed. Analytical determinations were performed independently on each batch.

For each treatment, T0 was defined as the time immediately after completion of the corresponding production or functionalization procedure. Thus, T0 corresponded to freshly produced oil for U_EVOO, to the oil obtained immediately after co-processing during malaxation for TG_MAL, and to the end of the 30-day infusion period and subsequent removal of the solid plant material for TG_INF. This definition was adopted because the aim of the study was to compare the final oils obtained through the two functionalization protocols. Therefore, TG_MAL was evaluated immediately after oil extraction and co-processing, whereas TG_INF was evaluated after completion of the 30-day infusion period, when the infused oil represented the final product obtained by this procedure. Consequently, T0 represents the starting point of storage for each finished product. This design also means that TG_INF had already undergone 30 days of additional processing before its storage baseline. Differences at the respective T0 values therefore include the effects of this additional contact time, as well as those of the incorporation route; they cannot be attributed to the route alone.

At the start of the respective storage periods (T0), U_EVOO, TG_MAL, and TG_INF were transferred into 100 mL dark glass bottles and stored at room temperature, protected from light, for 360 days. Samples were analyzed at T0 and after 15 (T15), 30 (T30), 60 (T60), 180 (T180), and 360 (T360) days of storage.

### 2.2. Quality Parameters and Color Evaluation

Free acidity (FA), peroxide value (PV), and UV spectrophotometric indices (K_232_, K_268_, and ΔK) were determined according to the official methods specified in the Commission Implementing Regulation (EU) 2022/2105, as amended [19]. Free acidity was expressed as a percentage of oleic acid (% oleic acid), whereas peroxide value was expressed as milliequivalents of active oxygen per kilogram of oil (meq O_2_ kg^−1^).

Color measurements were performed using a Konica Minolta CM-700d spectrophotometer (Konica Minolta, Osaka, Japan). Color was expressed according to the CIELAB color space using the L*, a*, and b* coordinates, where L* represents lightness, a* the red–green coordinate, and b* the yellow–blue coordinate. Color saturation was expressed as chroma (C*), calculated as follows:

$$C^{*}=\;\sqrt{{\left(a^{*}\right)}^{2}+{\left(b^{*}\right)}^{2}}$$

### 2.3. Spectrophotometric Estimation of Apparent Pigment Contents

Oil samples were diluted with n-hexane (1:1), and absorbance was measured at 670 and 470 nm to estimate apparent chlorophyll-derived and carotenoid contents, respectively. Results were expressed as mg kg^−1^ of pheophytin equivalents for the 670 nm estimate and mg kg^−1^ of lutein equivalents for the 470 nm estimate. These measurements do not separate individual pigments. In the functionalized oils, non-pigment chromophores from the spices may contribute to the measured absorbance; therefore, the estimates are operational spectrophotometric equivalents rather than chromatographically validated pigment concentrations.

### 2.4. Extraction of the Phenolic Fraction from Oil Samples

The phenolic fraction of the oil samples was extracted using a modified method based on Montedoro et al. [20]. Briefly, U_EVOO, TG_MAL, and TG_INF were subjected to liquid–liquid extraction with methanol/water (70:30, *v*/*v*) and n-hexane. After phase separation, the hydroalcoholic fraction containing the phenolic compounds was recovered, and the solvent was evaporated using a rotary evaporator (Heidolph WB 2000, Heidolph Instruments GmbH & Co. KG, Schwabach, Germany). The resulting residue was reconstituted in methanol/water (50:50, *v*/*v*), and the extracts were stored at −20 °C until analysis.

### 2.5. Determination of Phenolic Compounds

Total phenolic content (TPC) was determined using the Folin–Ciocalteu assay. Briefly, 10 mL of the sample was transferred into a 100 mL volumetric flask and mixed with 50 mL of ultrapure water, 5 mL of Folin–Ciocalteu reagent, and 20 mL of a 15% (*w*/*v*) sodium carbonate (Na_2_CO_3_) solution. The volume was then brought to 100 mL with ultrapure water. The mixture was allowed to react for 2 h in the dark at room temperature, after which absorbance was measured at 750 nm using an Agilent 8453 UV–Vis spectrophotometer (Agilent Technologies, Waldbronn, Germany). TPC was quantified using gallic acid as the reference standard and expressed as mg of gallic acid equivalents (GAE) per kg of oil.

### 2.6. Determination of α-Tocopherol Content

The α-tocopherol content of U_EVOO, TG_MAL, and TG_INF was determined by ultra-high-performance liquid chromatography coupled with fluorescence detection (UHPLC-FLD), according to Custureri et al. [11]. Briefly, oil samples were diluted with 2-propanol (1:10, *v*/*v*), vortex-mixed, and filtered through a 0.45 μm nylon syringe filter prior to chromatographic analysis. An aliquot of 5 μL was injected into the UHPLC system equipped with a fluorescence detector (RF-20A/RF-20Axs, Shimadzu Corporation, Kyoto, Japan).

Chromatographic separation was performed under isocratic conditions using methanol/acetonitrile (50:50, *v*/*v*) as the mobile phase at a flow rate of 0.5 mL min^−1^. Fluorescence detection was carried out at excitation and emission wavelengths of 290 and 330 nm, respectively. α-Tocopherol was identified and quantified by external calibration using an authentic α-tocopherol standard, and the results were expressed as mg kg^−1^ of oil.

### 2.7. Antioxidant Activity

The antioxidant activity of the extracts obtained from U_EVOO, TG_MAL, and TG_INF according to Section 2.4 was evaluated using complementary in vitro assays based on different antioxidant mechanisms.

Radical-scavenging activity was assessed using the ABTS [2,2′-azino-bis(3-ethylbenzothiazoline-6-sulfonic acid)] and DPPH (2,2-diphenyl-1-picrylhydrazyl) assays according to Sicari et al. [21]. Absorbance was measured at 734 nm for the ABTS assay, whereas the DPPH assay was performed under the experimental conditions described in the cited method, and the absorbance was measured at 515 nm. Ascorbic acid was used as the positive reference compound in both assays.

The ability of the extracts to inhibit lipid oxidation was evaluated using the β-carotene–linoleic acid bleaching assay in an oil-in-water emulsion according to Sicari et al. [21]. Absorbance was monitored at 470 nm, and antioxidant activity was evaluated based on the inhibition of β-carotene bleaching.

Ferric reducing antioxidant power was evaluated using the FRAP assay according to Sicari et al. [21].

All antioxidant assays were performed in triplicate. Results from the DPPH, ABTS, and β-carotene bleaching assays are expressed as IC_50_ values, whereas FRAP results are expressed according to the units specified for the corresponding assay.

### 2.8. Enzyme Inhibitory Activity

The in vitro inhibitory activity of the oil samples against enzymes involved in carbohydrate and lipid digestion was evaluated using α-amylase, α-glucosidase, and pancreatic lipase assays.

α-amylase and α-glucosidase inhibitory activities were determined according to Sicari et al. [21], with absorbance measured at 540 nm. Pancreatic lipase inhibitory activity was evaluated according to the same reference methodology, using orlistat as the positive control [21]. For all enzyme inhibition assays, results were expressed as IC_50_ values, defined as the sample concentration required to inhibit 50% of enzyme activity.

### 2.9. Sensory Evaluation

The sensory characteristics of U_EVOO, TG_MAL, and TG_INF were evaluated by a trained sensory panel composed of seven panelists. A quantitative descriptive analysis (QDA) was performed to characterize the sensory profile of the samples, using selected descriptors related to the typical sensory characteristics of olive oil and to the turmeric–ginger mixture.

Before evaluation, the panelists were informed that the study involved olive oils functionalized with turmeric and ginger. Because assessors were aware that the study involved turmeric- and ginger-functionalized oils, expectation effects on the perception of spice-related descriptors cannot be excluded. Visual, olfactory, and gustatory attributes were evaluated using a 9-point intensity scale, ranging from 1 (absence of the perceived attribute) to 9 (maximum perceived intensity).

For each sensory attribute, results were expressed as the mean score assigned by the panelists. The sensory profiles of the oils were graphically represented using radar plots.

### 2.10. Statistical Analysis

Results are expressed as mean ± standard deviation (SD) of three independent experimental batches (*n* = 3). Statistical analyses were performed using SPSS Statistics, version 22.0 (IBM Corp., Armonk, NY, USA). Data were analyzed by two-way analysis of variance (ANOVA) to evaluate the effects of treatment (U_EVOO, TG_MAL, and TG_INF), storage time, and their interaction. When significant effects were detected, mean comparisons were performed using Tukey’s multiple comparison test. Differences were considered statistically significant at *p* < 0.05.

## 3. Results and Discussion

### 3.1. Evolution of the Main Quality Parameters During Storage

The evolution of the main physicochemical quality parameters of U_EVOO, TG_MAL, and TG_INF during 360 days of storage is reported in Table 1. Overall, both the functionalization procedure and storage time affected the quality indices, although the magnitude and temporal pattern of these changes differed among the three oils.

Free acidity increased progressively during storage in all samples, with significant changes over time (*p* < 0.01). At T0, acidity values were 0.33% in U_EVOO, 0.28% in TG_MAL, and 0.51% in TG_INF. U_EVOO and TG_MAL did not differ significantly from each other, whereas TG_INF showed a significantly higher value (*p* < 0.05). This pattern persisted throughout storage, with TG_INF consistently showing the highest acidity. At T360, values reached 0.74% in U_EVOO, 0.68% in TG_MAL, and 1.04% in TG_INF, with the latter remaining significantly higher than the other two samples (*p* < 0.01). The lower acidity observed in TG_MAL compared with TG_INF suggests that incorporation of the turmeric–ginger mixture during malaxation did not promote hydrolytic deterioration to the same extent as the post-extraction infusion procedure. The higher acidity of TG_INF may be related to the prolonged contact of the oil with the powdered spices during the 30-day infusion period, although this interpretation should be considered cautiously because the present study did not directly investigate the mechanisms responsible for this increase. Hydrolysis of triacylglycerol ester bonds releases free fatty acids and requires water. Residual moisture carried by commercial powders or retained in dispersed aqueous microdomains could therefore facilitate hydrolysis during prolonged oil–spice contact. Lipolytic activity would be an additional possible contributor only if active enzymes were present and retained activity under these conditions. The powders were described as food-grade commercial products, so enzyme activity cannot be assumed by the plant species alone. In TG_MAL, the 40 min contact was followed by separation of the oil from vegetation water and solids, whereas TG_INF remained in contact with the powders for 30 days before filtration. These differences offer a plausible explanation for the higher initial acidity of TG_INF, but neither moisture nor lipase activity was measured, and their contributions cannot be distinguished. Moreover, powder removal after infusion means that subsequent storage changes cannot automatically be ascribed to continued contact with the bulk spices. At T180 and T360, TG_INF had mean acidity values of 0.81% and 1.04%, respectively, exceeding the 0.80% benchmark for unmodified EVOO. This is a quality limitation of the protocol under the tested conditions. The functionalized samples are olive oil-based products with added spices; Article 12(6) of Regulation (EU) 2022/2104 states that oils with other foodstuffs added must not bear the legal olive-oil category names [22]. Accordingly, comparison with the EVOO acidity benchmark does not establish the legal category of the functionalized product, and the present results do not support a claim that it retains EVOO status during storage.

A clearer differentiation among treatments was observed for peroxide value (PV), which increased significantly during storage in all samples (*p* < 0.01), indicating an overall increase in measured hydroperoxide burden. At T0, PV was 7.04 meq O_2_ kg^−1^ in U_EVOO, 5.47 meq O_2_ kg^−1^ in TG_MAL, and 6.50 meq O_2_ kg^−1^ in TG_INF. TG_MAL showed the lowest initial value and maintained the lowest PV throughout the storage period. At T360, PV reached 15.82 meq O_2_ kg^−1^ in U_EVOO, 9.79 meq O_2_ kg^−1^ in TG_MAL, and 14.03 meq O_2_ kg^−1^ in TG_INF, with significant differences among all three treatments (*p* < 0.01). Thus, the net increase in PV was smaller in TG_MAL than in the other oils. TG_INF also showed a lower final PV than U_EVOO, but the difference was less pronounced than that observed for TG_MAL. These findings indicate that the functionalization procedure strongly influenced the evolution of primary oxidation products and suggest that co-processing turmeric and ginger during malaxation was associated with a lower peroxide burden than post-extraction infusion during prolonged storage. Process-dependent differences in oxidative behavior have also been reported for olive oils functionalized with ginger or turmeric using malaxation and infusion approaches [11,12,13]. Changes in conventional quality parameters during the storage of olive oils flavored with herbs and spices have also been documented by Gambacorta et al. [23]. PV reflects the net accumulation of hydroperoxides, which depends on both their formation and decomposition [24]. A lower PV is therefore evidence of a lower measured hydroperoxide burden, rather than conclusive evidence of lower total oxidation or slower peroxide formation. The contribution of spice-derived antioxidants is plausible, but the different initial loadings per oil mass, processing times and uncharacterized compositions prevent identification of the protective constituents or isolation of a single process mechanism.

The UV spectrophotometric indices showed a more complex pattern. For K_232_, U_EVOO did not exhibit significant changes over storage, with values ranging from 2.10 to 2.36. In contrast, significant time-dependent variations were observed for both functionalized oils (*p* < 0.01). TG_MAL showed a relatively high initial K_232_ value (3.18), which decreased to 1.96 at T30 and subsequently fluctuated, reaching 2.46 at T360. TG_INF started at 1.79, remained comparatively low up to T180, and increased to 2.31 at T360. Differences among treatments were significant at several storage times, but not at T30 or T360. Therefore, unlike PV, K_232_ did not show a simple progressive increase during storage and did not provide evidence of a consistent treatment-related pattern over the entire experimental period.

For K_268_, U_EVOO remained relatively stable throughout storage, ranging from 0.19 to 0.24, with no significant effect of storage time. TG_MAL showed higher values than U_EVOO during most of the storage period, although its temporal variation was limited. TG_INF displayed a different behavior: K_268_ remained between 0.21 and 0.27 from T0 to T60 and then increased markedly to 0.46 at T180 and 0.45 at T360 (*p* < 0.01). At the final storage time, TG_MAL and TG_INF showed significantly higher K_268_ values than U_EVOO. In unmodified olive oil, K_268_ is used as an indicator associated with conjugated trienes and some secondary oxidation products. In these spice-containing matrices, however, the measured absorbance need not originate exclusively from oxidation. These results therefore suggest that the effects of functionalization on oxidative evolution cannot be inferred solely from peroxide value. Despite the lower PV observed in TG_MAL, its K_268_ values were generally higher than those of U_EVOO, whereas TG_INF showed a pronounced increase during the later stages of storage. PV and UV extinction indices measure different chemical responses. Hydroperoxide decomposition can reduce or limit PV while conjugated or carbonyl-containing products develop; conversely, UV-active botanical constituents can increase extinction without a corresponding increase in hydroperoxides. In TG_MAL, K_268_ changed from 0.48 at T0 to 0.50 at T15, 0.36 at T30–T60, 0.48 at T180 and 0.40 at T360. This non-monotonic pattern may reflect changing contributions from oxidation products and spice-derived chromophores or their transformation products. Aromatic and conjugated constituents may contribute to the UV region, while curcuminoids also contribute strongly to the visible region. The curcumin spectrum depends on the solvent and chemical environment [25]; a spectrum measured for a pure standard under different conditions cannot quantitatively correct these mixed-oil measurements. Without matrix-matched spectra and chromatographic profiling, the contribution at 232 or 268 nm cannot be assigned or quantified. Thus, the K_268_ fluctuations should not be interpreted as confirmed episodes of either improved stability or accelerated oxidation.

ΔK remained close to zero in most samples and storage times. No significant temporal variation was observed for U_EVOO or TG_MAL, whereas TG_INF showed a significant effect of storage time (*p* < 0.01), mainly attributable to the value observed at T15 (0.07 ± 0.03). Differences among treatments were otherwise not significant, except at T15 (*p* < 0.05). The generally low ΔK values and the absence of a consistent increasing trend indicate that this parameter was comparatively stable during storage.

Taken together, the quality parameters indicate that the response to turmeric–ginger functionalization depended on both the analytical index and the production procedure. TG_MAL was characterized by the lowest peroxide values throughout storage and by a lower final acidity than TG_INF, indicating a lower measured accumulation of hydroperoxides. However, the behavior of K_232_ and K_268_ was not fully parallel to that of PV, highlighting both the different chemical endpoints and the possibility of matrix-dependent UV contributions. TG_INF showed the highest acidity throughout the experiment and a marked increase in K_268_ during the later storage stages. These results therefore indicate that co-processing during malaxation and post-extraction infusion differently affected the evolution of the conventional quality indices and that the oxidative behavior of the oils should be interpreted by considering the different analytical parameters together rather than individually. The influence of the flavoring procedure on the evolution of quality parameters during storage has also been investigated by Cecchi et al. [26], who compared co-milling and post-extraction infusion in chili pepper-flavored olive oils.

### 3.2. Evolution of CIELAB Color Parameters During Storage

The evolution of the CIELAB color parameters of U_EVOO, TG_MAL, and TG_INF during 360 days of storage is reported in Table 2. Significant differences were observed among treatments and over storage time, indicating that both the functionalization procedure and storage influenced the color characteristics of the oils.

Lightness (L*) changed significantly over time in all three samples (*p* < 0.01). At T0, L* values were 33.70 for U_EVOO, 34.30 for TG_MAL, and 32.68 for TG_INF. TG_MAL showed the highest initial value, whereas TG_INF was significantly darker than TG_MAL, with U_EVOO showing an intermediate value. During the early stages of storage, L* decreased in all samples, reaching minimum values at T30 of 32.40, 31.20, and 28.80 for U_EVOO, TG_MAL, and TG_INF, respectively. Thereafter, lightness progressively increased, and at T360, values reached 36.50 in U_EVOO, 36.80 in TG_MAL, and 33.60 in TG_INF. From T15 onwards, TG_INF consistently showed significantly lower L* values than the other two oils (*p* < 0.01), whereas U_EVOO and TG_MAL did not differ significantly. These results indicate that post-extraction infusion produced a persistently darker oil, whereas co-processing during malaxation resulted in a lightness profile more similar to that of untreated EVOO.

The a* coordinate, representing the green–red axis, also showed significant changes during storage (*p* < 0.01). At T0, a* values were 3.42, 3.08, and 2.84 for U_EVOO, TG_MAL, and TG_INF, respectively, with significant differences among all treatments. Values initially decreased, reaching their lowest levels around T30, and subsequently increased. At T360, a* reached 4.10 in U_EVOO, 3.78 in TG_MAL, and 3.18 in TG_INF. U_EVOO generally maintained the highest a* values, while TG_INF showed the lowest values throughout storage. The persistence of significant differences among treatments indicates that the two functionalization procedures differently affected the red–green component of oil color.

A different pattern was observed for the b* coordinate, which represents the blue–yellow axis. Significant treatment and storage effects were evident throughout the experiment (*p* < 0.01). At T0, TG_INF showed the highest b* value (6.65), followed by U_EVOO (6.38) and TG_MAL (5.60), with significant differences among the three oils. During the first 30 days, b* increased in all samples, although the increase was particularly marked in TG_INF, which reached 8.40 at T30. Thereafter, b* progressively decreased, reaching 5.35, 4.75, and 6.35 at T360 for U_EVOO, TG_MAL, and TG_INF, respectively. Despite this decline, TG_INF maintained significantly higher b* values throughout storage, whereas TG_MAL generally showed the lowest values. This behavior indicates that post-extraction infusion produced a more pronounced yellow component than co-processing during malaxation and untreated EVOO.

The differences observed for b* were also reflected in chroma (C*), which expresses color saturation. At T0, C* values were similar for U_EVOO (7.24) and TG_INF (7.23), whereas TG_MAL showed a significantly lower value (6.39). During storage, TG_INF exhibited the highest color saturation, reaching a maximum C* of 8.55 at T30. U_EVOO also reached its maximum at T30 (7.66), whereas TG_MAL reached its highest value earlier, at T15 (7.06). Subsequently, chroma decreased in all samples. At T360, C* values were 6.74 in U_EVOO, 6.09 in TG_MAL, and 7.11 in TG_INF, with TG_INF and U_EVOO not significantly different from each other, whereas TG_MAL showed the lowest value. Overall, TG_INF displayed greater color saturation during most of the storage period, largely reflecting its consistently higher b* values.

The distinct color profiles observed for TG_MAL and TG_INF may be related to differences in the transfer and subsequent evolution of pigments and other colored compounds derived from both the olive matrix and the turmeric–ginger mixture. In particular, turmeric contains intensely colored curcuminoids, while olive oil color is mainly influenced by chlorophyll and carotenoid pigments. The extent to which these compounds are transferred, transformed, or degraded may depend on the functionalization procedure and storage conditions. Previous studies on spice-functionalized olive oils have similarly shown that turmeric, ginger, and other botanical materials can modify color parameters and that the magnitude of these changes depends on the processing method and storage time [11,12,13]. During co-processing, transferred compounds can partition among oil, vegetation water and solid material before separation, while infusion exposes an already separated lipid phase to the powders for 30 days. The resulting chromophore mixtures may therefore differ even with the same nominal powder percentage [9,13]. The higher b* and lower L* of TG_INF are compatible with a stronger contribution of yellow spice-derived constituents, but color coordinates neither identify curcuminoids nor quantify their recovery. Changes after powder removal may involve redistribution, transformation or degradation of dissolved or colloidally dispersed constituents, which were not individually measured.

The CIELAB results demonstrate that the two functionalization procedures generated clearly distinguishable color profiles. TG_INF was characterized by lower lightness and a more pronounced yellow component during storage, together with generally higher color saturation. In contrast, TG_MAL showed L* values closer to those of U_EVOO but lower b* and C* values. These differences confirm that the visual characteristics of turmeric–ginger-functionalized oils depend strongly on the method used to incorporate the botanical ingredients and evolve significantly during prolonged storage.

### 3.3. Evolution of Apparent Spectrophotometric Pigment Estimates During Storage

The evolution of the spectrophotometrically estimated apparent chlorophyll and carotenoid contents of U_EVOO, TG_MAL, and TG_INF during 360 days of storage is reported in Table 3. Significant differences were observed among treatments and over storage time, with the two functionalization procedures producing markedly different optical profiles.

At T0, the apparent chlorophyll content was similar in U_EVOO and TG_INF, with values of 13.21 and 13.20 mg kg^−1^, respectively, whereas TG_MAL showed a significantly lower value of 5.51 mg kg^−1^ (*p* < 0.01). The three oils subsequently followed different temporal patterns. In U_EVOO, apparent chlorophyll content remained relatively stable during the first 180 days, with values ranging from 12.99 to 14.21 mg kg^−1^, before decreasing significantly to 11.01 mg kg^−1^ at T360. TG_MAL showed an initial increase, reaching 11.10 mg kg^−1^ at T30, followed by a progressive decrease to 5.11 mg kg^−1^ at T360. In contrast, TG_INF exhibited a marked increase during the early and intermediate stages of storage, reaching 21.10 mg kg^−1^ at T15, 21.81 mg kg^−1^ at T30, and a maximum of 23.01 mg kg^−1^ at T60. Thereafter, the apparent chlorophyll content decreased to 19.00 mg kg^−1^ at T180 and 13.31 mg kg^−1^ at T360. From T15 onwards, TG_INF consistently showed significantly higher values than both U_EVOO and TG_MAL (*p* < 0.01).

The different optical profiles do not establish differences in the concentrations of individual chlorophylls or carotenoids. The major analytical concern at 470 nm is possible overlap with the broad visible absorption of turmeric-derived curcuminoids. Curcumin has an environment-dependent visible spectrum [25], so overlap at the carotenoid measurement wavelength is plausible even though its main absorption maximum is not necessarily at 470 nm. This does not justify assuming an identical interference at 670 nm, which is farther from its usual main visible band. Other co-extracted constituents, transformation products, light scattering or baseline effects nevertheless cannot be excluded without matrix validation. The lack of chromatographic pigment separation is therefore a significant limitation: the 470 and 670 nm results should be interpreted as apparent optical equivalents, not validated specific pigment contents.

Apparent carotenoid content also varied significantly according to treatment and storage time. At T0, U_EVOO and TG_INF showed identical values of 6.10 mg kg^−1^, whereas TG_MAL had a significantly lower apparent carotenoid content of 5.00 mg kg^−1^ (*p* < 0.05). During the early stages of storage, apparent carotenoid estimates increased in all three oils, although the magnitude of the increase differed substantially among treatments. U_EVOO reached a maximum of 8.40 mg kg^−1^ at T30, while TG_MAL reached 7.80 mg kg^−1^ at T15 and remained relatively high at T30 (7.50 mg kg^−1^). The largest increase was observed in TG_INF, which reached 9.00 mg kg^−1^ at T15 and a maximum of 11.60 mg kg^−1^ at T30.

After these early maxima, apparent carotenoid estimates generally decreased. At T360, values were 4.80 mg kg^−1^ in both U_EVOO and TG_MAL and 6.40 mg kg^−1^ in TG_INF. Thus, at the end of storage, TG_INF retained a significantly higher apparent carotenoid content than the other two oils (*p* < 0.01), whereas U_EVOO and TG_MAL did not differ significantly from each other. Overall, TG_INF showed the highest apparent carotenoid estimates from T15 onwards, with significant differences among treatments at all storage times.

The higher apparent pigment levels observed in TG_INF during much of the storage period may be related to the post-extraction contact between the oil and the turmeric–ginger powders. Turmeric contains strongly colored compounds that may contribute to the optical properties of the functionalized oil and potentially interfere with or contribute to spectrophotometric pigment estimates. Conversely, during co-processing, the botanical material is added to the olive paste before oil separation, and the subsequent extraction process may limit the transfer or retention of some pigment-like compounds in the final oil. Process-dependent effects on the color and pigment characteristics of spice-functionalized olive oils have also been reported in previous studies using ginger and turmeric [11,12,13].

The pigment results also provide useful context for the CIELAB color changes reported in Table 2. TG_INF, which showed the highest apparent carotenoid content during most of the storage period, was also characterized by consistently higher b* values, indicating a more pronounced yellow component. This association is compatible with the contribution of yellow-colored compounds to the visual characteristics of the infused oil. Nevertheless, because the present spectrophotometric approach does not allow individual pigments or turmeric-derived chromophores to be distinguished, a direct causal relationship between specific compounds and CIELAB coordinates cannot be established.

Overall, the two functionalization procedures produced markedly different apparent pigment profiles. Post-extraction infusion resulted in higher apparent chlorophyll and carotenoid contents during most of the storage period, whereas TG_MAL generally showed the lowest apparent chlorophyll estimates and carotenoid values closer to those of U_EVOO by the end of storage. The non-linear temporal patterns, particularly the initial increases observed for several pigment measurements, indicate that the evolution of the spectrophotometrically estimated pigment fraction cannot be interpreted solely in terms of pigment degradation. Targeted chromatographic analysis would be required to distinguish individual olive pigments from turmeric- and ginger-derived colored compounds and to clarify their respective evolution during storage. Because the bulk spice solids were removed before TG_INF storage, the increases at T15–T60 cannot simply be explained as continued extraction from the original powder suspension. They may involve residual dispersed material, transformations or analytical matrix effects, but the present measurements do not resolve these alternatives. In particular, an increase in the apparent chlorophyll estimate is not evidence of chlorophyll synthesis or enrichment during storage.

### 3.4. Evolution of α-Tocopherol and Total Phenolic Content During Storage

The evolution of α-tocopherol and total phenolic content (TPC) in U_EVOO, TG_MAL, and TG_INF during 360 days of storage is reported in Table 4. Both parameters changed significantly during storage, although the effects of the functionalization procedure differed between α-tocopherol and the phenolic fraction.

At T0, α-tocopherol content was 388.41 mg kg^−1^ in U_EVOO, 353.41 mg kg^−1^ in TG_MAL, and 383.41 mg kg^−1^ in TG_INF. U_EVOO showed the highest initial value, although it did not differ significantly from TG_INF, whereas TG_MAL showed a significantly lower concentration (*p* < 0.05). Similar differences were observed during the early stages of storage. At T15 and T30, TG_MAL remained significantly lower than the other two oils, while U_EVOO and TG_INF showed comparable α-tocopherol levels. At T60, T180, and T360, however, differences among treatments were no longer statistically significant.

A progressive decline in α-tocopherol was observed in all three oils during storage (*p* < 0.01). In U_EVOO, α-tocopherol decreased from 388.41 mg kg^−1^ at T0 to 318.41 mg kg^−1^ at T360, corresponding to a reduction of approximately 18%. TG_MAL decreased from 353.41 to 303.41 mg kg^−1^, corresponding to a reduction of approximately 14%, whereas TG_INF decreased from 383.41 to 333.41 mg kg^−1^, corresponding to a reduction of approximately 13%. Thus, although TG_MAL had the lowest absolute α-tocopherol concentration throughout most of the storage period, the relative decrease from T0 to T360 was slightly smaller than that observed in U_EVOO. TG_INF showed the highest numerical α-tocopherol concentration at T360 and a similar relative retention to TG_MAL; however, the absence of significant differences among treatments at the final storage times does not support attributing a statistically greater α-tocopherol preservation to either functionalization procedure.

The progressive decrease in α-tocopherol is consistent with its role as a lipid-phase antioxidant and with its consumption during oxidative processes occurring during prolonged oil storage. Nevertheless, the evolution of α-tocopherol did not directly parallel that of peroxide value. In particular, TG_MAL showed the lowest peroxide values throughout storage despite having lower absolute α-tocopherol concentrations than U_EVOO and TG_INF. This finding suggests that the lower accumulation of primary oxidation products observed in TG_MAL cannot be explained solely by its α-tocopherol content and may involve the contribution of other endogenous or spice-derived constituents. Similar interactions among tocopherols, phenolic compounds, and other minor constituents have been considered in previous studies dealing with the oxidative behavior of conventional and spice-functionalized olive oils [11,12,13]. The initial α-tocopherol differences may involve recovery of the endogenous oil fraction and oxidative exposure during processing. However, this study did not establish a tocopherol mass balance or quantify tocopherol in the powders, so neither a spice-derived addition nor processing-related consumption can be assigned as the cause of the lower initial value in TG_MAL. Interpretation should remain at the level of the measured concentration changes.

A different pattern was observed for TPC. At T0, U_EVOO showed a TPC of 453.16 mg kg^−1^, whereas significantly higher values were found in both functionalized oils: 554.65 mg kg^−1^ in TG_MAL and 514.65 mg kg^−1^ in TG_INF (*p* < 0.01). TG_MAL showed the highest initial TPC and differed significantly from both TG_INF and U_EVOO. At T15, the same ranking was maintained, with values of 569.65, 529.65, and 462.65 mg kg^−1^ for TG_MAL, TG_INF, and U_EVOO, respectively. At T30, TG_MAL and TG_INF showed comparable values, both significantly higher than U_EVOO. This pattern was maintained from T60 onwards, with the two functionalized oils belonging to the same statistical group and U_EVOO showing significantly lower TPC values.

TPC decreased significantly during prolonged storage in all samples (*p* < 0.01), particularly after T60. At T360, values had declined to 309.65 mg kg^−1^ in U_EVOO, 379.65 mg kg^−1^ in TG_MAL, and 384.65 mg kg^−1^ in TG_INF. Therefore, both functionalized oils retained significantly higher TPC than U_EVOO at the end of storage (*p* < 0.01), whereas the small numerical difference between TG_INF and TG_MAL was not statistically significant. Relative to their respective T0 values, the decreases were approximately 32% for U_EVOO, 32% for TG_MAL, and 25% for TG_INF. The latter therefore showed a numerically smaller proportional decline in TPC, although the final concentrations of TG_INF and TG_MAL were statistically comparable.

The higher TPC observed in the functionalized oils indicates that the incorporation of turmeric and ginger modified the pool of Folin–Ciocalteu-reactive compounds present in the oil. Both spices are sources of phenolic constituents, including curcuminoids in turmeric and gingerols, shogaols, and related compounds in ginger, which may contribute directly or indirectly to the measured response. The initially higher TPC of TG_MAL suggests that co-processing during malaxation favored the presence of Folin-reactive compounds in the freshly obtained functionalized oil. In contrast, the smaller proportional decrease observed in TG_INF resulted in very similar TPC values for the two functionalized oils after prolonged storage. Previous studies on ginger- and turmeric-functionalized olive oils have likewise reported that the enrichment procedure and storage can influence the phenolic fraction and its evolution over time [11,12,13]. The initially higher Folin response of TG_MAL is compatible with process-dependent recovery of reducing compounds but cannot be interpreted as a higher extraction yield because the powder loading was defined on olive paste rather than on the recovered oil mass. Partitioning among aqueous, solid and oil phases during co-processing differs from direct oil contact during infusion and may produce qualitatively different extracts [9,13].

It should also be considered that the Folin–Ciocalteu assay provides an estimate of the overall reducing capacity of the extracted fraction rather than a selective quantification of individual phenolic compounds. Consequently, the higher TPC measured in TG_MAL and TG_INF cannot be attributed to specific olive, turmeric, or ginger phenolics on the basis of the present data alone. Targeted chromatographic analysis would be required to determine the contribution and storage evolution of individual compounds. The Folin–Ciocalteu reagent also reacts with some non-phenolic reducing substances [27], and results expressed as gallic acid equivalents do not provide curcuminoid or gingerol concentrations. Accordingly, the reported TPC is an operational measure of Folin-reactive material and cannot demonstrate the retention or degradation of a particular spice marker.

The results show that turmeric–ginger functionalization affected α-tocopherol and TPC differently. Functionalization did not result in higher α-tocopherol concentrations than U_EVOO at the beginning of storage, and no significant differences among treatments were observed from T60 onwards. Conversely, both TG_MAL and TG_INF showed consistently higher TPC than U_EVOO, with the difference persisting up to T360. TG_MAL exhibited the highest TPC during the initial storage period, whereas TG_INF and TG_MAL showed statistically comparable values during the later stages of storage. These findings further indicate that the different behavior of oils during storage cannot be attributed to a single antioxidant component but likely reflect the combined contribution and evolution of several minor constituents.

### 3.5. Inhibitory Activity Against α-Amylase, α-Glucosidase, and Pancreatic Lipase During Storage

The inhibitory activities of U_EVOO, TG_MAL, and TG_INF against α-amylase, α-glucosidase, and pancreatic lipase during 360 days of storage are reported in Table 5. Results are expressed as IC_50_ values; therefore, lower values indicate greater inhibitory activity. Significant differences among treatments and storage times were observed for all three enzymes (*p* < 0.01), although the magnitude and temporal evolution of the effects differed according to the enzymatic target. The observed effects refer exclusively to in vitro enzyme inhibition and should not be interpreted as evidence of physiological or therapeutic effects.

At T0, α-amylase inhibition was markedly greater in the functionalized oils than in U_EVOO. U_EVOO showed an IC_50_ of 269.02 µg mL^−1^, whereas TG_MAL and TG_INF showed substantially lower values of 135.30 and 135.37 µg mL^−1^, respectively. During storage, IC_50_ values generally increased in both functionalized oils, indicating a progressive reduction in α-amylase inhibitory activity. The IC_50_ of TG_MAL increased from 135.30 µg mL^−1^ at T0 to 286.09 µg mL^−1^ at T360, whereas that of TG_INF increased from 135.37 to 259.61 µg mL^−1^. U_EVOO showed a less regular temporal pattern, increasing to 345.31 µg mL^−1^ at T60, subsequently decreasing to 240.29 µg mL^−1^ at T180, and reaching 289.32 µg mL^−1^ at T360. At the end of storage, TG_INF showed a significantly lower IC_50_ than U_EVOO and TG_MAL (*p* < 0.01), whereas U_EVOO and TG_MAL did not differ significantly from each other. Thus, the marked initial enhancement of α-amylase inhibition associated with both functionalization procedures progressively diminished during storage, although TG_INF retained significantly greater inhibitory activity at T360. Acarbose, used as the positive control, showed an IC_50_ of 50.18 µg mL^−1^. Previous studies have similarly reported that spice functionalization can modify the in vitro α-amylase inhibitory activity of olive oils and its evolution during storage [11,12].

A more distinct pattern was observed for α-glucosidase inhibition. At T0, U_EVOO showed an IC_50_ of 137.34 µg mL^−1^, whereas TG_MAL and TG_INF showed significantly lower and virtually identical values of 125.83 and 125.84 µg mL^−1^, respectively. IC_50_ values increased significantly with storage time in all three oils, but the magnitude of the increase differed markedly among treatments. U_EVOO showed the greatest loss of inhibitory activity, reaching 337.56 µg mL^−1^ at T60, 587.49 µg mL^−1^ at T180, and 778.23 µg mL^−1^ at T360. In comparison, TG_MAL reached 321.10 µg mL^−1^ at T360, whereas TG_INF showed the lowest final IC_50_, 223.18 µg mL^−1^. From T30 onwards, TG_INF consistently exhibited significantly lower IC_50_ values than TG_MAL, while both functionalized oils remained significantly more active than U_EVOO. Therefore, post-extraction infusion was associated with the greatest persistence of α-glucosidase inhibitory activity during prolonged storage. Acarbose, used as the positive control, showed an IC_50_ of 35.57 µg mL^−1^.

Marked differences were also observed for pancreatic lipase inhibition. At T0, U_EVOO showed an IC_50_ of 143.46 µg mL^−1^, whereas both TG_MAL and TG_INF showed the same markedly lower value of 55.02 µg mL^−1^. Thus, turmeric–ginger functionalization was associated with substantially greater initial pancreatic lipase inhibitory activity. IC_50_ values increased significantly during storage in all samples, indicating a progressive reduction in inhibitory activity. At T180, values reached 253.81 µg mL^−1^ in U_EVOO, 127.68 µg mL^−1^ in TG_MAL, and 100.09 µg mL^−1^ in TG_INF. At T360, the corresponding values were 312.97, 176.70, and 140.21 µg mL^−1^, respectively. At this final storage time, significant differences were observed among all three oils (*p* < 0.01), with TG_INF showing the greatest inhibitory activity, followed by TG_MAL and U_EVOO. Notably, the IC_50_ of TG_INF at T360 (140.21 µg mL^−1^) remained close to that of fresh U_EVOO at T0 (143.46 µg mL^−1^), indicating substantial persistence of the functionalization-associated effect despite the decline observed during storage. Orlistat, used as the positive control, showed an IC_50_ of 37.44 µg mL^−1^. Similar effects on pancreatic lipase inhibition have previously been reported in spice-functionalized olive oils [11].

The different behavior observed among the three enzymatic assays indicates that the effect of turmeric–ginger functionalization was strongly dependent on the enzymatic target. For α-amylase, both functionalized oils showed markedly greater inhibition at the beginning of storage, but the differences progressively narrowed, and at T360 only TG_INF remained significantly more active than the other samples. In contrast, the functionalization-associated differences were maintained more clearly for α-glucosidase and pancreatic lipase, particularly in TG_INF. At T360, TG_INF showed significantly lower IC_50_ values than both TG_MAL and U_EVOO for all three enzymes, although the magnitude of this advantage differed substantially among the assays.

The differences in enzyme-inhibitory activity may reflect the composition and storage evolution of the constituents recovered in the tested extracts. Experimental studies on isolated compounds provide a basis for considering turmeric- and ginger-derived constituents as possible contributors. Bisdemethoxycurcumin isolated from *Curcuma longa* inhibited human pancreatic α-amylase, with an uncompetitive inhibition mechanism when starch was used as the substrate [28]. Du et al. [29] also demonstrated α-glucosidase inhibition by natural curcuminoids, with bisdemethoxycurcumin exhibiting non-competitive inhibition. Furthermore, isolated 6-gingerol and 6-shogaol inhibited α-amylase and α-glucosidase in a study of compounds obtained from *Aframomum melegueta* [30]. For pancreatic lipase, He et al. [31] experimentally confirmed the inhibitory activity of curcumin, demethoxycurcumin, and bisdemethoxycurcumin, reporting IC_50_ values of 0.52 ± 0.04, 1.12 ± 0.05, and 3.30 ± 0.08 mg mL^−1^, respectively. Enzyme kinetics indicated reversible competitive inhibition, while spectroscopic analyses supported interactions with pancreatic lipase. These results demonstrate that structurally related curcuminoids can differ in inhibitory potency and provide a basis for considering them as possible contributors to the activity of turmeric-containing extracts. However, curcuminoid concentrations were not determined in the present study; therefore, the observed pancreatic lipase inhibition cannot be attributed specifically to these compounds.

The treatment-dependent differences in enzyme inhibition may be related to the distinct conditions governing the transfer of botanical constituents into the oils. During co-processing, turmeric and ginger interact with the olive paste for 40 min, and released constituents can partition between the oil, vegetation water, and solid material before separation. In contrast, infusion involves direct contact between the spice powders and the extracted oil for 30 days, potentially allowing progressive transfer of oil-soluble constituents and compositional changes during contact. The two procedures may therefore yield different constituent profiles in the oils and, subsequently, in the hydroalcoholic extracts used for enzyme assays.

The inhibitory responses suggest that these differences were enzyme-dependent. TG_MAL and TG_INF showed nearly identical initial IC_50_ values, particularly for α-amylase and pancreatic lipase, but their responses diverged during storage. For α-amylase, the treatment difference remained comparatively limited, whereas TG_INF showed lower α-glucosidase IC_50_ values than TG_MAL from T30 onwards and greater residual pancreatic lipase inhibition at T360. These patterns are consistent with differences in the composition and evolution of constituents capable of interacting with each enzyme, rather than a uniform increase in inhibitory activity across all targets. Furthermore, the statistically comparable TPC values of TG_MAL and TG_INF at T360, despite their different enzyme-inhibitory activities, indicate that total phenolic content alone cannot account for the effect of the functionalization procedure. The greater residual activity of TG_INF should therefore not be interpreted as proof of greater preservation of a specific curcuminoid or gingerol. Because individual constituents were not quantified, the relative contributions of selective transfer, transformation, and persistence remain unresolved. Finally, T0 followed 30 days of infusion for TG_INF but coincided with completion of oil production for TG_MAL; thus, the comparison includes differences in both the incorporation environment and treatment duration.

The turmeric–ginger functionalization was associated with greater in vitro inhibition of the digestive enzymes investigated, although the magnitude and persistence of the effect depended strongly on both the enzyme and the functionalization procedure. Both TG_MAL and TG_INF showed pronounced initial effects against α-amylase and pancreatic lipase, while differences in α-glucosidase inhibition became progressively more evident during storage. After 360 days, TG_INF showed the lowest IC_50_ values for α-amylase, α-glucosidase, and pancreatic lipase, indicating the greatest persistence of the in vitro enzyme-inhibitory activity among the investigated oils. These findings should nevertheless be interpreted strictly within the context of the in vitro assays and cannot be directly extrapolated to physiological, nutritional, or therapeutic effects.

### 3.6. Antioxidant Activity During Storage

**Figure 1:**
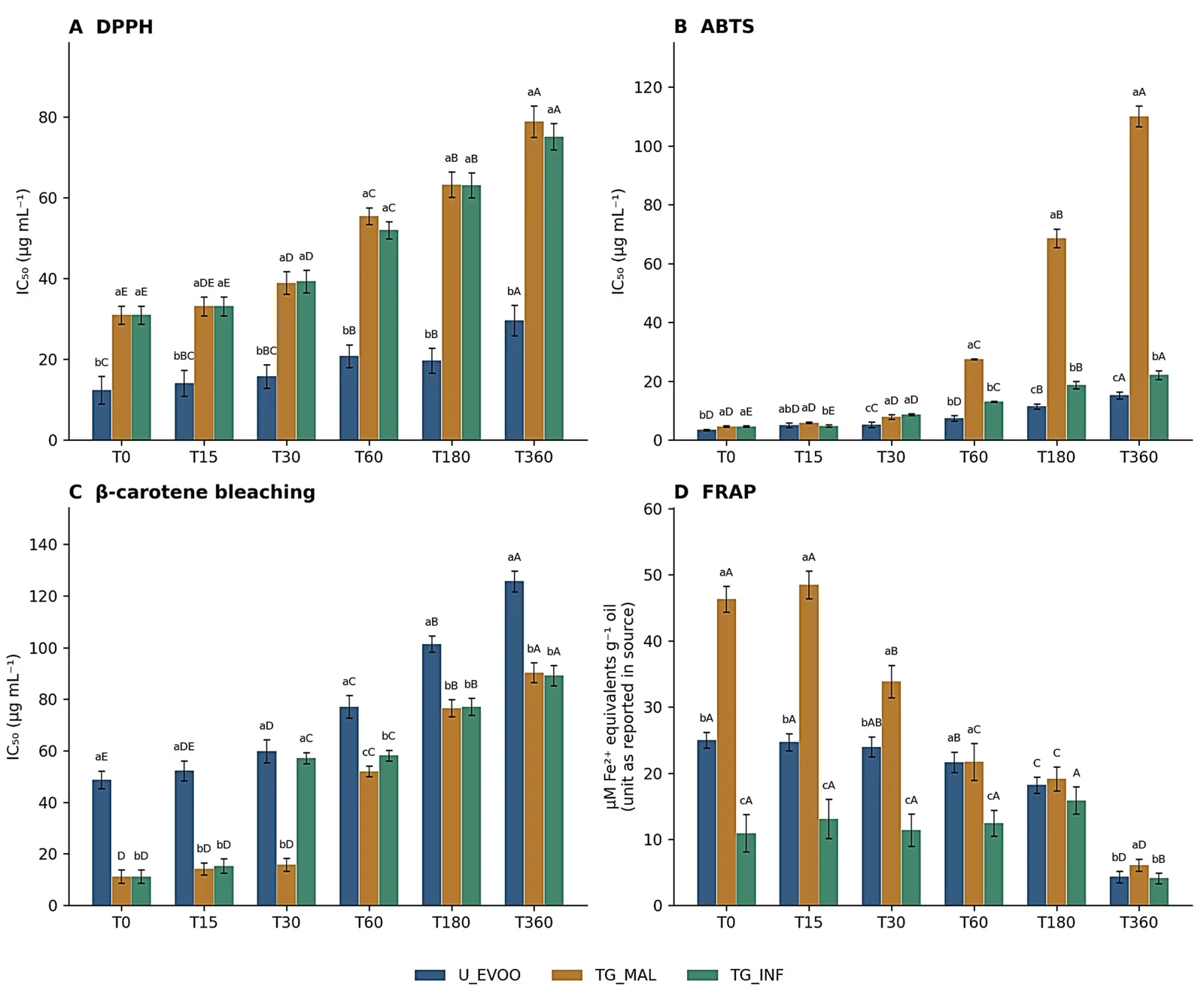
Changes in antioxidant assay responses during storage. Panels (**A**–**C**) show DPPH, ABTS and β-carotene bleaching IC_50_ values (µg mL^−1^), respectively; lower values indicate stronger apparent activity. Panel (**D**) shows FRAP in the units reported for the source data; higher values indicate a greater reducing response. Bars show means and error bars show standard deviations, as supplied in the embedded chart (n = 3, in accordance with the manuscript). Letters reproduce the original chart annotations without reanalysis. Within each assay, different lowercase letters indicate differences among treatments at the same storage time, and different uppercase letters indicate differences among storage times within the same treatment, according to the statistical annotation supplied with the source chart.

The antioxidant activity of extracts obtained from U_EVOO, TG_MAL, and TG_INF during 360 days of storage was evaluated using DPPH, ABTS, β-carotene bleaching, and FRAP assays (Figure 1).

Overall, the results showed a strongly assay-dependent response, indicating that turmeric–ginger functionalization had different effects on radical-scavenging activity, protection against lipid peroxidation, and ferric reducing capacity. This finding is consistent with the different reaction mechanisms and sensitivities of the antioxidant assays and highlights the importance of using complementary methods when assessing the antioxidant properties of olive oil and functionalized oils.

In the DPPH assay, U_EVOO showed the greatest radical-scavenging activity throughout storage, as indicated by its lower IC_50_ values compared with the functionalized oils. At T0, the IC_50_ of U_EVOO was 12.33 µg mL^−1^, whereas both TG_MAL and TG_INF showed values of approximately 30.91 µg mL^−1^. During storage, DPPH IC_50_ values increased in all samples, indicating a progressive reduction in radical-scavenging capacity. At T360, values reached 29.54 µg mL^−1^ in U_EVOO, 78.83 µg mL^−1^ in TG_MAL, and 75.11 µg mL^−1^ in TG_INF. Thus, turmeric–ginger functionalization did not improve DPPH radical-scavenging activity, and U_EVOO maintained the strongest response even after prolonged storage.

A similar overall trend was observed in the ABTS assay. At T0, U_EVOO showed an IC_50_ of 3.43 µg mL^−1^, compared with approximately 4.58 µg mL^−1^ for both TG_MAL and TG_INF. Antioxidant activity subsequently decreased during storage, although the extent of this decline differed markedly among treatments. At T360, U_EVOO showed an IC_50_ of 15.21 µg mL^−1^, whereas TG_MAL reached 110.03 µg mL^−1^ and TG_INF 22.10 µg mL^−1^. Therefore, the loss of ABTS radical-scavenging activity was particularly pronounced in TG_MAL, while TG_INF showed substantially greater persistence of activity. Nevertheless, U_EVOO maintained the lowest final IC_50_ and therefore the greatest ABTS radical-scavenging capacity at T360.

The β-carotene bleaching assay provided a markedly different response. At T0, U_EVOO showed an IC_50_ of 48.72 µg mL^−1^, whereas TG_MAL and TG_INF showed much lower values of 11.13 and 11.12 µg mL^−1^, respectively. Since lower IC_50_ values in this assay indicate a greater ability to protect β-carotene against oxidative degradation, the extracts from both functionalized oils displayed markedly greater initial activity in the lipid peroxidation model. During storage, IC_50_ values progressively increased in all samples, indicating a decline in protective activity. At T360, U_EVOO reached 125.63 µg mL^−1^, whereas TG_MAL and TG_INF showed values of 90.31 and 89.15 µg mL^−1^, respectively. Thus, although the protective effect decreased substantially during storage, the extracts from both functionalized oils maintained a numerically greater capacity to counteract β-carotene bleaching than the extracts from U_EVOO at the end of the experiment. Unlike DPPH and ABTS radical-scavenging assays, the β-carotene–linoleic acid bleaching assay evaluates antioxidant protection in an oil-in-water emulsion. In this heterogeneous system, antioxidant effectiveness depends on both chemical reactivity and polarity-dependent distribution between the aqueous phase, lipid domains, and oil–water interface. Consequently, antioxidant protection depends on the effective concentration and reactivity of compounds near the oxidizing lipid substrate, rather than on their total concentration alone [32].

The FRAP assay further confirmed the assay-dependent effect of functionalization. At T0, TG_MAL showed the highest ferric reducing capacity, with a value of 46.29 µM Fe^2+^ equivalents g^−1^ oil, compared with 25.01 µM Fe^2+^ equivalents g^−1^ oil for U_EVOO and 10.93 µM Fe^2+^ equivalents g^−1^ oil for TG_INF. TG_MAL maintained a high reducing capacity at T15, reaching 48.46 µM Fe^2+^ equivalents g^−1^ oil. Thereafter, FRAP values decreased markedly during storage in all samples. At T360, the values were 4.31 µM Fe^2+^ equivalents g^−1^ oil in U_EVOO, 6.09 µM Fe^2+^ equivalents g^−1^ oil in TG_MAL, and 4.09 µM Fe^2+^ equivalents g^−1^ oil in TG_INF. Therefore, co-processing during malaxation produced the strongest initial enhancement of ferric reducing capacity, although this advantage progressively diminished during prolonged storage.

The contrasting responses obtained with the different antioxidant assays are particularly relevant when considered together with the compositional results. Both functionalized oils showed higher TPC than U_EVOO throughout storage, yet this did not translate into greater DPPH or ABTS radical-scavenging activity. Conversely, TG_MAL and TG_INF showed substantially greater initial protection in the β-carotene bleaching assay, while TG_MAL exhibited the highest initial FRAP value. These findings indicate that the antioxidant behavior of the extracts cannot be predicted solely from the total phenolic content measured in the corresponding oils.

Turmeric-derived curcuminoids and ginger-derived gingerols and shogaols contain hydrophobic structural regions together with polar oxygen-containing groups. If recovered in the tested extracts, these constituents could redistribute between the aqueous phase, lipid domains, and oil–water interface upon addition to the β-carotene–linoleic acid emulsion. Compounds with an appropriate balance of lipophilic and hydrophilic properties may attain effective concentrations near the oxidizing lipid substrate, whereas more hydrophilic constituents may remain predominantly in the aqueous phase. Such a distribution could contribute to the greater protection observed for the extracts from TG_MAL and TG_INF despite their weaker DPPH and ABTS responses. This interpretation is consistent with the interfacial explanation of the polar paradox [33]; however, greater lipophilicity does not invariably increase antioxidant effectiveness, as demonstrated by the cutoff effect in emulsified systems [34]. The extraction procedure determines which constituents are recovered and subsequently available in the assays; therefore, these results characterize the antioxidant behavior of the extracted fractions rather than the complete antioxidant system of the original oils. Since individual spice-derived compounds, their extraction recoveries, and their distributions within the assay emulsion were not determined, the proposed mechanism remains a plausible interpretation.

The stronger DPPH and ABTS responses of extracts from U_EVOO indicate greater radical-scavenging activity under the specific conditions of these assays, rather than necessarily greater protection against lipid oxidation. The superior performance of U_EVOO in these assays may reflect differences in the composition and reactivity of the constituents accessible in the reaction medium, where solvent-dependent solubility, reaction kinetics, and accessibility of reactive groups influence the measured response. Therefore, the contrasting assay rankings cannot be explained by total phenolic content or polarity alone. FRAP measures an additional complementary property, namely ferric reducing capacity.

Differences between TG_MAL and TG_INF may also arise from the distinct extraction environments and contact times associated with the two functionalization procedures. During co-processing, turmeric and ginger constituents interact with a heterogeneous olive paste containing oil, vegetation water, and solids for 40 min, and their recovery in the final oil depends on release from the botanical material, partitioning between phases, and retention in the aqueous or solid fractions during separation. Conversely, infusion involves prolonged contact with the extracted oil for 30 days, which may favor the progressive transfer of oil-soluble constituents while also allowing compositional changes during contact. These different conditions could therefore produce distinct antioxidant profiles even when the same spice mixture is used [9]. The higher initial TPC and FRAP values of TG_MAL are consistent with greater recovery of Folin-reactive and ferric-reducing constituents, but do not establish which compounds were responsible. Conversely, the lower ABTS IC_50_ of TG_INF compared with TG_MAL at T360 suggests better retention of constituents reactive under the ABTS assay conditions. The similar β-carotene bleaching responses of the two treatments indicate that these compositional differences did not produce equally pronounced differences in protection within the emulsion model. Moreover, because the assays were performed on hydroalcoholic extracts, the measured responses reflect the constituents recovered by the analytical extraction rather than all compounds present in the oils. Targeted compositional analysis would be required to distinguish differences in compound transfer, transformation, and persistence between the two procedures.

Overall, turmeric–ginger functionalization produced a selective rather than generalized enhancement of antioxidant activity. The extract from U_EVOO maintained the strongest DPPH and ABTS radical-scavenging activity, whereas the extracts from both functionalized oils showed greater initial protection in the β-carotene bleaching model and retained numerically lower IC_50_ values than the extract from U_EVOO at T360. TG_MAL exhibited the highest initial ferric reducing capacity, but FRAP activity declined markedly during storage. Among the two functionalization procedures, TG_INF showed greater persistence of ABTS activity than TG_MAL, while their β-carotene bleaching activities were very similar at the end of storage. These results demonstrate that the antioxidant effect of turmeric–ginger functionalization depends strongly on both the functionalization procedure and the analytical system considered.

### 3.7. Sensory Profile of Turmeric–Ginger Functionalized Olive Oils

**Figure 2:**
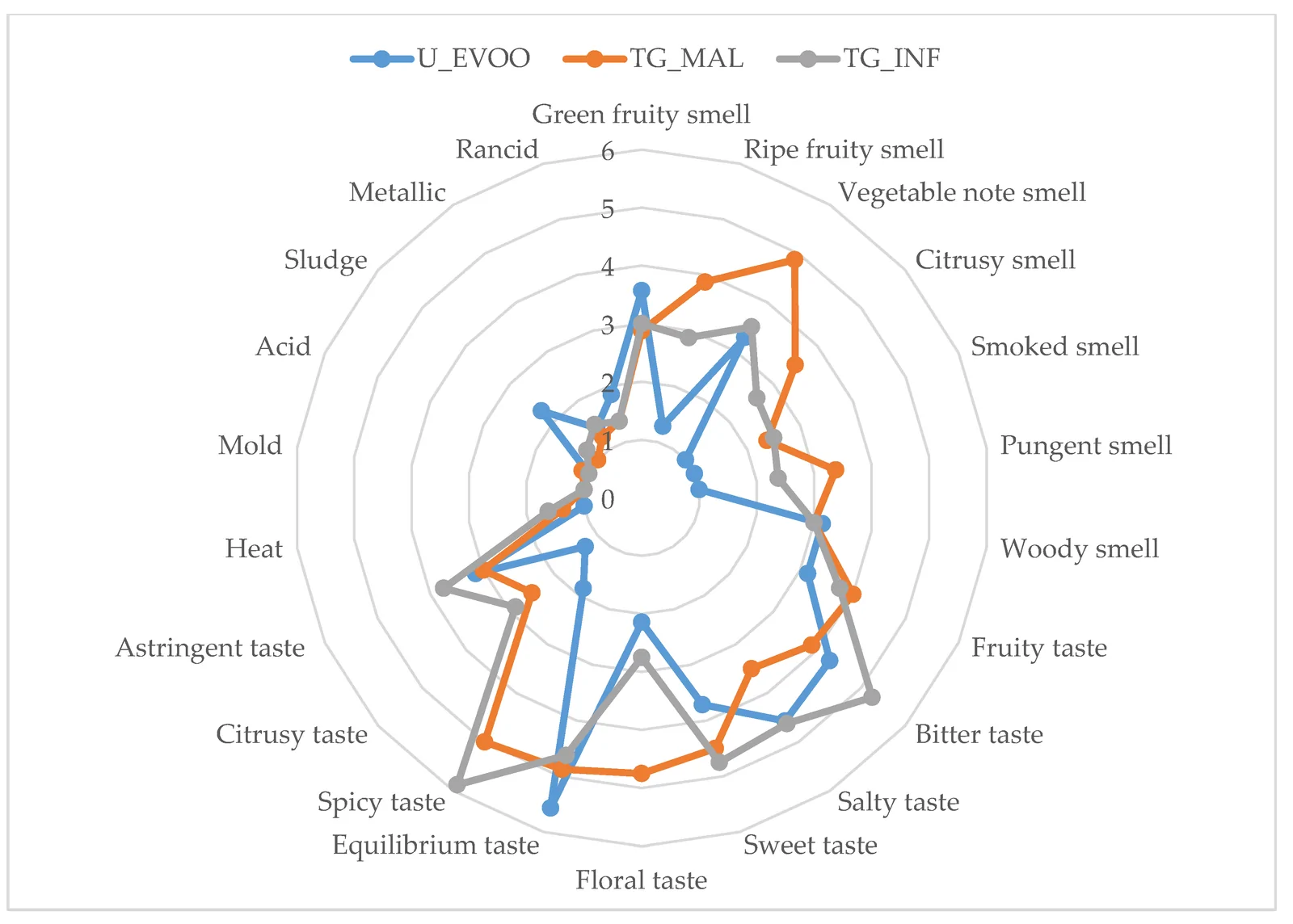
Radar plot showing the quantitative descriptive sensory profile of extra virgin olive oil (U_EVOO) and oils functionalized with turmeric and ginger (TG) obtained by co-processing during malaxation (TG_MAL) and post-extraction infusion (TG_INF). Sensory attributes were assessed by a trained panel using a 9-point intensity scale (1 = absent; 9 = extremely perceptible). Values represent the mean intensity scores for each sensory descriptor.

Sensory analysis showed distinct olfactory and gustatory profiles among U_EVOO, TG_MAL, and TG_INF (Figure 2). Overall, the main differences concerned fruity and aromatic notes, as well as bitterness, spiciness, and astringency. Similar process-dependent variations in the sensory characteristics of spice-functionalized olive oils have previously been reported [9,11,13]. The influence of storage time on the sensory, chemical, and physical characteristics of flavored olive oils has also been investigated by Kacalova and Jarosova [35].

Regarding olfactory attributes, U_EVOO showed the highest green fruity score (3.57), compared with 2.88 for TG_MAL and 3.00 for TG_INF. In contrast, TG_MAL showed higher scores for several other aromatic descriptors, including ripe fruity (3.88), vegetable (4.88), citrusy (3.50), and pungent (3.38) notes. TG_INF generally showed intermediate values for these attributes. Smoked odor was slightly more pronounced in both functionalized oils, whereas woody notes were similar among the three samples. These results indicate that TG_MAL was characterized by a broader aromatic profile than U_EVOO and TG_INF.

Differences were also evident in the gustatory profile. Fruity taste increased from 3.14 in U_EVOO to 4.00 in TG_MAL and 3.75 in TG_INF. The most pronounced differences concerned spicy taste, which increased from 1.86 in U_EVOO to 5.00 in TG_MAL and 5.88 in TG_INF. TG_INF also showed the highest bitterness (5.25) and astringency (3.75), whereas TG_MAL showed the highest floral score (4.75). Citrus and sweet sensations were also higher in both functionalized oils than in U_EVOO. Similar modifications in fruity, bitter, pungent, and aromatic attributes have been observed in olive oils functionalized with ginger, turmeric, and other botanical ingredients [9,11,13].

The equilibrium descriptor was highest in U_EVOO (5.57), followed by TG_MAL (4.88) and TG_INF (4.63). Negative descriptors, including mold, acid, metallic, sludge, and rancid notes, remained generally low in all samples. However, since the sensory evaluation was descriptive rather than hedonic, differences among the oils should not be interpreted in terms of consumer preference or overall acceptability.

Overall, the sensory profiles differed according to the functionalization protocol. TG_MAL showed higher intensities of several aromatic descriptors, particularly ripe fruity, vegetable, citrusy, pungent, and floral notes, whereas TG_INF was characterized by more pronounced bitter, spicy, and astringent sensations. These results are consistent with previous studies showing that different spice-enrichment procedures are associated with distinct sensory profiles in functionalized olive oils [9,11,13]. Differences in transfer and retention of volatile and non-volatile spice constituents during the brief contact with olive paste versus prolonged contact with the separated oil provide a plausible basis for the different aroma and taste profiles [9,13]. However, volatile profiles and individual pungent or bitter compounds were not measured, so sensory descriptors cannot be assigned to particular molecules. The assessors were informed that turmeric–ginger oils were being evaluated, creating the possibility of expectation bias, especially for spice-related attributes, as prior information about product characteristics can influence sensory expectations and perception, as reported by Piqueras-Fiszman and Spence [36]. Even if samples were coded, prior disclosure of the ingredients would not eliminate this source of bias. The observed profiles should therefore be treated as descriptive responses under an informed evaluation condition, rather than unbiased estimates of the isolated sensory effect of each process. Future evaluation should withhold ingredient and treatment information, randomize serving order and compare informed with blinded assessments where feasible.

## 4. Conclusions

Turmeric–ginger functionalization yielded olive oil-based products with distinct quality, compositional, in vitro functional, and sensory characteristics over 360 days of storage. The differences depended on the preparation protocol and the analytical endpoint; neither protocol was consistently superior across all parameters.

Co-processing during malaxation (TG_MAL) was associated with the lowest peroxide values throughout storage (9.79 meq O_2_ kg^−1^ at T360, versus 14.03 for TG_INF and 15.82 for U_EVOO), indicating a lower measured hydroperoxide burden. This finding does not by itself establish lower overall oxidation, particularly because the UV extinction indices did not follow the same pattern and may include contributions from spice-derived chromophores. Post-extraction infusion (TG_INF) resulted in the highest free acidity (1.04% at T360), exceeding the 0.80% benchmark applicable to unmodified EVOO. The functionalized products should not be assumed to retain the legal EVOO designation.

Both functionalized oils maintained higher total phenolic contents than U_EVOO at the end of storage (379.65 mg kg^−1^ for TG_MAL and 384.65 mg kg^−1^ for TG_INF, versus 309.65 mg kg^−1^ for U_EVOO), without a significant difference between the functionalized treatments at T360. α-Tocopherol decreased during storage, with no significant between-treatment differences from T60 onwards. Antioxidant activity varied across analytical methods: neither functionalized oil consistently outperformed U_EVOO in DPPH and ABTS assays, whereas both showed lower apparent IC_50_ values in the β-carotene bleaching assay. The latter result warrants caution because absorbance at 470 nm may be influenced by turmeric-derived constituents.

In vitro digestive-enzyme inhibition generally weakened during storage, but TG_INF retained the lowest IC_50_ values for α-amylase, α-glucosidase, and pancreatic lipase at T360. These responses indicate greater residual activity under the assay conditions, not demonstrated physiological or therapeutic benefits. Sensory analysis identified distinct descriptive profiles, with TG_MAL exhibiting stronger fruity, vegetable, citrusy, pungent, and floral attributes and TG_INF more pronounced bitter, spicy, and astringent notes. The descriptive design and prior disclosure of the spice ingredients do not permit conclusions about consumer preference.

The comparison reflects complete preparation protocols rather than isolated incorporation mechanisms: the nominal spice additions were calculated on different receiving matrices, and TG_INF underwent an additional 30-day infusion before its T0 storage measurement. Furthermore, spectrophotometric pigment estimates and some activity assays may be affected by matrix interference, while the individual spice-derived compounds responsible for the observed effects were not quantified. Targeted chromatographic profiling, controlled dose-matched process comparisons, and studies of bioaccessibility, reproducibility, and consumer acceptance are therefore needed before broader technological or nutritional claims can be made.

## Figures and Tables

**Table 1:** Changes in the main quality parameters of extra virgin olive oil (U_EVOO) and oils functionalized with turmeric and ginger (TG) obtained by co-processing during malaxation (TG_MAL) and post-extraction infusion (TG_INF) over 360 days of storage.

Acidity	T0	T15	T30	T60	T180	T360	Sign.
U_EVOO	0.33 ± 0.03 ^bC^	0.35 ± 0.03 ^bC^	0.37 ± 0.03 ^bC^	0.41 ± 0.03 ^bC^	0.53 ± 0.03 ^bD^	0.74 ± 0.04 ^bE^	**
TG_MAL	0.28 ± 0.02 ^bC^	0.27 ± 0.01 ^bC^	0.29 ± 0.01 ^bC^	0.34 ± 0.02 ^bC^	0.47 ± 0.02 ^bD^	0.68 ± 0.03 ^bE^	**
TG_INF	0.51 ± 0.06 ^aC^	0.55 ± 0.06 ^aC^	0.59 ± 0.06 ^aBC^	0.66 ± 0.05 ^aBC^	0.81 ± 0.05 ^aB^	1.04 ± 0.06 ^aA^	**
Sign.	*	*	**	**	**	**	
Peroxide Value	T0	T15	T30	T60	T180	T360	Sign.
U_EVOO	7.04 ± 0.15 ^aE^	7.40 ± 0.22 ^aDE^	8.13 ± 0.23 ^aD^	9.24 ± 0.29 ^aC^	12.13 ± 0.28 ^aB^	15.82 ± 0.29 ^aA^	**
TG_MAL	5.47 ± 0.12 ^bC^	5.69 ± 0.20 ^bC^	5.53 ± 0.13 ^cC^	6.08 ± 0.12 ^cC^	7.60 ± 0.20 ^cB^	9.79 ± 0.21 ^cA^	**
TG_INF	6.50 ± 0.20 ^aD^	6.32 ± 0.20 ^bD^	6.71 ± 0.23 ^bCD^	7.49 ± 0.17 ^bC^	10.01 ± 0.23 ^bB^	14.03 ± 0.27 ^bA^	**
Sign.	**	**	**	**	**	**	
ΔK	T0	T15	T30	T60	T180	T360	Sign.
U_EVOO	−0.003 ± 0.000	−0.003 ± 0.000 ^ab^	−0.003 ± 0.000	−0.003 ± 0.000	−0.001 ± 0.000	0.00 ± 0.00	ns
TG_MAL	−0.03 ± 0.04	−0.01 ± 0.01 ^b^	−0.01 ± 0.00	0.00 ± 0.01	0.00 ± 0.00	0.00 ± 0.00	ns
TG_INF	−0.003 ± 0.00 ^B^	0.07 ± 0.03 ^aA^	0.00 ± 0.00 ^B^	0.00 ± 0.00 ^B^	0.00 ± 0.00 ^B^	0.00 ± 0.00 ^B^	**
Sign.	ns	*	ns	ns	ns	ns	
K232	T0	T15	T30	T60	T180	T360	Sign.
U_EVOO	2.18 ± 0.08 ^b^	2.19 ± 0.08 a	2.10 ± 0.06	2.14 ± 0.05 ^ab^	2.30 ± 0.06 ^a^	2.36 ± 0.11	ns
TG_MAL	3.18 ± 0.02 ^aA^	2.15 ± 0.02 ^aCD^	1.96 ± 0.01 ^D^	2.33 ± 0.09 ^aBC^	2.05 ± 0.04 ^bD^	2.46 ± 0.1 ^B^	**
TG_INF	1.79 ± 0.02 ^cBC^	1.82 ± 0.04 ^bBC^	2.02 ± 0.06 ^B^	1.99 ± 0.02 ^bBC^	1.75 ± 0.04 ^cC^	2.31 ± 0.12 ^A^	**
Sign.	**	*	ns	*	**	ns	
K268	T0	T15	T30	T60	T180	T360	Sign.
U_EVOO	0.23 ± 0.01 ^b^	0.23 ± 0.02 ^b^	0.19 ± 0.02 ^b^	0.19 ± 0.02 ^b^	0.22 ± 0.01 ^b^	0.24 ± 0.01 ^b^	ns
TG_MAL	0.48 ± 0.09 ^a^	0.5 ± 0.04 ^a^	0.36 ± 0.02 ^a^	0.36 ± 0.02 ^a^	0.48 ± 0.03 ^a^	0.4 ± 0.02 ^a^	*
TG_INF	0.21 ± 0.01 ^bB^	0.27 ± 0.01 ^bB^	0.24 ± 0.02 ^bB^	0.26 ± 0.02 ^bB^	0.46 ± 0.02 ^aA^	0.45 ± 0.01 ^aA^	**
Sign.	*	**	**	*	**	**	

Free acidity (% oleic acid), peroxide value (meq O_2_ kg^−1^ oil), specific extinction coefficients (K232 and K268), and ΔK were determined according to the official analytical methods for virgin olive oils. Results are expressed as mean ± standard deviation (*n* = 3). Statistical differences among treatments and storage times were evaluated by two-way ANOVA followed by Tukey’s post hoc test (*p* ≤ 0.05), as indicated by the significance rows and columns (“Sign”). Statistical significance is reported as follows: ns = not significant; * *p* < 0.05; ** *p* < 0.01. Different lowercase letters within the same column indicate significant differences among samples at the same storage time. Different uppercase letters within the same row indicate significant differences among storage times within the same sample.

**Table 2:** Changes in the CIELAB color parameters of extra virgin olive oil (U_EVOO) and oils functionalized with turmeric and ginger (TG) obtained by co-processing during malaxation (TG_MAL) and post-extraction infusion (TG_INF) during 360 days of storage.

L*	T0	T15	T30	T60	T180	T360	Sign.
U_EVOO	33.7 ± 1.41 abBC	33.3 ± 1.25 aBC	32.4 ± 1.15 aC	33 ± 1.3 aBC	34.6 ± 1.55 aAB	36.5 ± 1.9 aA	**
TG_MAL	34.3 ± 0.72 aBC	32.6 ± 1.05 aCD	31.2 ± 1.2 aD	32.8 ± 1.35 aCD	34.9 ± 1.65 aB	36.8 ± 1.95 aA	**
TG_INF	32.68 ± 1.23 bAB	30.2 ± 1.2 bCD	28.8 ± 1.35 bD	29.4 ± 1.45 bD	31.5 ± 1.7 bBC	33.6 ± 2 bA	**
Sign.	*	**	**	**	**	**	
a*	T0	T15	T30	T60	T180	T360	Sign.
U_EVOO	3.42 ± 0.07 aBC	3.3 ± 0.13 aC	3 ± 0.16 aD	3.15 ± 0.19 aCD	3.62 ± 0.27 aB	4.1 ± 0.36 aA	**
TG_MAL	3.08 ± 0.11 bB	2.5 ± 0.15 bC	2.05 ± 0.18 bD	2.65 ± 0.22 bC	3.22 ± 0.3 bB	3.78 ± 0.4 bA	**
TG_INF	2.84 ± 0.1 cA	2.1 ± 0.17 cC	1.58 ± 0.2 bD	1.82 ± 0.24 cCD	2.45 ± 0.32 cB	3.18 ± 0.42 cA	**
Sign.	**	**	**	**	**	**	
b*	T0	T15	T30	T60	T180	T360	Sign.
U_EVOO	6.38 ± 0.09 bBC	6.2 ± 0.15 cCD	7.05 ± 0.2 bA	6.6 ± 0.22 bB	6.05 ± 0.28 bD	5.35 ± 0.35 bE	**
TG_MAL	5.6 ± 0.14 cBC	6.6 ± 0.2 bA	6.3 ± 0.22 cA	5.85 ± 0.24 cB	5.3 ± 0.3 cC	4.75 ± 0.36 cD	**
TG_INF	6.65 ± 0.16 aD	7.6 ± 0.22 aB	8.4 ± 0.25 aA	7.75 ± 0.28 aB	7.05 ± 0.33 aC	6.35 ± 0.4 aD	**
Sign.	**	**	**	**	**	**	
C*	T0	T15	T30	T60	T180	T360	Sign.
U_EVOO	7.24 ± 0.1 aB	7.02 ± 0.11 bBC	7.66 ± 0.17 bA	7.31 ± 0.23 bB	7.05 ± 0.29 bBC	6.74 ± 0.47 aC	**
TG_MAL	6.39 ± 0.13 bBCD	7.06 ± 0.19 bA	6.63 ± 0.24 cB	6.43 ± 0.22 cBC	6.21 ± 0.25 cCD	6.09 ± 0.31 bD	**
TG_INF	7.23 ± 0.16 aC	7.89 ± 0.22 aB	8.55 ± 0.25 aA	7.96 ± 0.29 aB	7.47 ± 0.38 aC	7.11 ± 0.47 aC	**
Sign.	**	**	**	**	**	**	

Color coordinates were expressed as L* (lightness), a* (green–red axis), b* (blue–yellow axis), and chroma (C*). Results are presented as mean ± standard deviation *(n* = 3). Statistical differences among treatments and storage times were determined by two-way ANOVA followed by Tukey’s post hoc test (*p* ≤ 0.05), as indicated by the significance rows and columns (“Sign”). Statistical significance is reported as follows: ns = not significant; * *p* < 0.05; ** *p* < 0.01. Different lowercase letters within the same column indicate significant differences among samples at the same storage time. Different uppercase letters within the same row indicate significant differences among storage times within the same sample.

**Table 3:** Changes in spectrophotometrically estimated apparent chlorophyll and carotenoid contents of extra virgin olive oil (U_EVOO) and oils functionalized with turmeric and ginger (TG) obtained by co-processing during malaxation (TG_MAL) and post-extraction infusion (TG_INF) during 360 days of storage. The 670 nm estimates are expressed as mg pheophytin equivalents kg^−1^ oil and the 470 nm estimates as mg lutein equivalents kg^−1^ oil. Values are apparent spectrophotometric equivalents; possible spice-derived spectral contributions have not been chromatographically resolved.

Apparent Chlorophyll Derivatives (mg Pheophytin Equivalents kg^−1^)	T0	T15	T30	T60	T180	T360	Sign.
U_EVOO	13.21 ± 0.22 ^aB^	12.99 ± 0.21 ^bB^	13.62 ± 0.28 ^bAB^	14.21 ± 0.22 ^bA^	13.31 ± 0.23 ^bAB^	11.01 ± 0.23 ^bC^	**
TG_MAL	5.51 ± 0.19 ^bCD^	8.2 ± 0.26 ^cB^	11.1 ± 0.3 ^cA^	6.41 ± 0.3 ^cC^	5.61 ± 0.23 ^cCD^	5.11 ± 0.23 ^cD^	**
TG_INF	13.2 ± 0.27 ^aD^	21.1 ± 0.46 ^aB^	21.81 ± 0.5 ^aAB^	23.01 ± 0.47 ^aA^	19 ± 0.45 ^aC^	13.31 ± 0.32 ^aD^	**
Sign.	**	**	**	**	**	**	
Apparent Carotenoids (mg Lutein Equivalents kg^−1^)	T0	T15	T30	T60	T180	T360	Sign.
U_EVOO	6.1 ± 0.2 ^aB^	6.1 ± 0.25 ^bB^	8.4 ± 0.33 ^bA^	6.9 ± 0.25 ^bB^	6.5 ± 0.27 ^aB^	4.8 ± 0.2 ^bC^	**
TG_MAL	5 ± 0.2 ^bC^	7.8 ± 0.31 ^aA^	7.5 ± 0.27 ^bA^	6.2 ± 0.23 ^bB^	5.2 ± 0.25 ^bBC^	4.8 ± 0.24 ^bC^	**
TG_INF	6.1 ± 0.25 ^aE^	9 ± 0.34 ^aB^	11.6 ± 0.4 ^aA^	8 ± 0.3 ^aBC^	7.5 ± 0.31 ^aCD^	6.4 ± 0.31 ^aDE^	**
Sign.	*	**	**	**	**	**	

Values are presented as mean ± standard deviation (*n* = 3). Different letters indicate significant differences among treatments and storage times according to two-way ANOVA followed by Tukey’s post hoc test (*p* ≤ 0.05). Statistical significance is reported as follows: ns = not significant; * *p* < 0.05; ** *p* < 0.01.

**Table 4:** Changes in α-tocopherol (α-Toc) and total phenolic content (TPC) of extra virgin olive oil (U_EVOO) and oils functionalized with turmeric and ginger (TG) obtained by co-processing during malaxation (TG_MAL) and post-extraction infusion (TG_INF) during 360 days of storage.

α-Toc (mg kg^−1^)	T0	T15	T30	T60	T180	T360	Sign.
U_EVOO	388.41 ± 7.35 aA	381.41 ± 7.64 bA	376.41 ± 7.92 bAB	368.41 ± 7.78 AB	348.41 ± 8.63 BC	318.41 ± 8.91 C	**
TG_MAL	353.41 ± 7.64 bA	349.41 ± 7.35 aA	346.41 ± 7.92 aA	340.41 ± 7.78 AB	328.41 ± 7.64 AB	303.41 ± 7.92 B	**
TG_INF	383.41 ± 8.06 abA	380.41 ± 8.34 aA	377.41 ± 8.2 aA	372.41 ± 8.49 A	358.41 ± 9.05 AB	333.41 ± 9.19 B	**
Sign.	*	*	*	ns	ns	ns	
TPC (mg kg^−1^)	T0	T15	T30	T60	T180	T360	Sign.
U_EVOO	453.16 ± 4.84 cA	462.65 ± 5.23 cA	456.65 ± 6.22 cA	439.65 ± 5.94 cA	384.65 ± 7.21 cB	309.65 ± 6.51 cC	**
TG_MAL	554.65 ± 6.22 aA	569.65 ± 7.35 aA	562.65 ± 8.63 aA	554.65 ± 12.3 aA	474.65 ± 9.33 aB	379.65 ± 8.34 aC	**
TG_INF	514.65 ± 5.94 bA	529.65 ± 6.79 bA	536.65 ± 7.92 aA	521.65 ± 8.34 aA	464.65 ± 8.49 aB	384.65 ± 8.06 aC	**
Sign.	**	**	**	**	**	**	

Results are expressed as mean ± standard deviation (*n* = 3). Statistical differences among treatments and storage times were evaluated by two-way ANOVA followed by Tukey’s post hoc test (*p* ≤ 0.05), as indicated by the significance rows and columns (“Sign”). Statistical significance is reported as follows: ns = not significant; * *p* < 0.05; ** *p* < 0.01. Different lowercase letters within the same column indicate significant differences among samples at the same storage time. Different uppercase letters within the same row indicate significant differences among storage times within the same sample.

**Table 5:** Changes in the inhibitory activity against α-amylase, α-glucosidase, and pancreatic lipase of extra virgin olive oil (U_EVOO) and oils functionalized with turmeric and ginger (TG) obtained by co-processing during malaxation (TG_MAL) and post-extraction infusion (TG_INF) during 360 days of storage.

α-Amylase	T0	T15	T30	T60	T180	T360	Sign
U_EVOO	269.02 ± 3.77 ^aD^	275.21 ± 3.85 ^aD^	303.38 ± 3.92 ^aB^	345.31 ± 4.05 ^aA^	240.29 ± 3.87 ^aE^	289.32 ± 4.90 ^aC^	**
TG_MAL	135.30 ± 3.56 ^bC^	138.57 ± 3.67 ^bC^	141.16 ± 3.87 ^cC^	224.84 ± 3.99 ^bB^	218.77 ± 3.28 ^bB^	286.09 ± 3.56 ^aA^	**
TG_INF	135.37 ± 3.65 ^aE^	141.23 ± 3.80 ^bE^	173.13 ± 3.44 ^bD^	210.64 ± 3.35 ^cC^	222.45 ± 3.81 ^bB^	259.61 ± 4.09 ^bA^	**
Sign	**	**	**	**	**	**	
Acarbose	50.18 ± 1.32						
α-Glucosidase	T0	T15	T30	T60	T180	T360	Sign
U_EVOO	137.34 ± 3.73 aA	145.18 ± 3.79 aB	198.81 ± 3.82 aC	337.56 ± 3.90 aD	587.49 ± 3.56 aE	778.23 ± 4.67 aE	**
TG_MAL	125.83 ± 3.45 bA	128.72 ± 3.22 bB	154.65 ± 3.09 bC	184.59 ± 3.01 bD	229.14 ± 2.66 bE	321.10 ± 3.07 bE	**
TG_INF	125.84 ± 3.05 bA	134.09 ± 3.66 bB	143.63 ± 3.12 cC	158.31 ± 3.01 cD	189.05 ± 3.22 cE	223.18 ± 3.87 cF	**
Sign	**	**	**	**	**	**	
Acarbose	35.57 ± 0.99						
Lipase	T0	T15	T30	T60	T180	T360	Sign
U_EVOO	143.46 ± 4.85 aA	155.52 ± 4.87 aB	173.43 ± 4.91 aC	206.54 ± 5.01 aD	253.81 ± 4.81 aE	312.97 ± 5.44 aF	**
TG_MAL	55.02 ± 1.09 bA	59.17 ± 1.19 bB	61.14 ± 1.22 bC	98.64 ± 1.02 bD	127.68 ± 1.35 bD	176.70 ± 1.78 bE	**
TG_INF	55.02 ± 4.09 bD	59.45 ± 4.16 bCD	66.97 ± 4.22 bC	95.75 ± 4.02 bB	100.09 ± 4.34 cB	140.21 ± 4.61 cA	**
Sign	**	**	**	**	**	**	
Orlistat	37.44 ± 1.08						

Enzyme inhibitory activity is expressed as IC_50_ (µg mL^−1^). Acarbose and Orlistat were used as positive controls for α-amylase/α-glucosidase and lipase inhibition, respectively. Data are presented as mean ± standard deviation (*n* = 3). Statistical differences among treatments and storage times were evaluated by two-way ANOVA followed by Tukey’s post hoc test (*p* ≤ 0.05), as indicated by the significance rows and columns (“Sign”). Statistical significance is reported as follows: ns = not significant; ** *p* < 0.01. Different lowercase letters within the same column indicate significant differences among samples at the same storage time. Different uppercase letters within the same row indicate significant differences among storage times within the same sample.

## Data Availability

The original contributions presented in the study are included in the article. Further inquiries can be directed to the corresponding authors.
